# Ganglioside-monosialic acid (GM1) for prevention of chemotherapy-induced peripheral neuropathy: a meta-analysis with trial sequential analysis

**DOI:** 10.1186/s12885-021-08884-4

**Published:** 2021-11-02

**Authors:** Shaoyong Wu, Xiaohui Bai, Caixia Guo, Zhimei Huang, Handong Ouyang, Jingxiu Huang, Weian Zeng

**Affiliations:** 1 Department of Anesthesiology, Sun Yat-Sen University Cancer Center, State Key Laboratory of Oncology in South China, Collaborative Innovation Center for Cancer Medicine, Guangzhou, Guangdong 510060 P. R. China; 2 Department of Anesthesiology, Guangdong Provincial Key Laboratory of Malignant Tumor Epigenetics and Gene Regulation, Sun Yat-sen Memorial Hospital, Sun Yat-sen University, Guangzhou, Guangdong 510289 P. R. China; 3 Department of Obstetrics, The Fourth Affiliated Hospital of Guangzhou Medical University (Zengcheng District People’s Hospital), Guangzhou, Guangdong 511300 P. R. China; 4 Department of Minimal Invasive Intervention, Sun Yat-sen University Cancer Center, State Key Laboratory of Oncology in South China, Collaborative Innovation Center for Cancer Medicine, Guangzhou, Guangdong 510060 P. R. China

**Keywords:** Ganglioside-monosialic acid, Chemotherapy, Neuropathy, meta-analysis

## Abstract

**Background:**

Chemotherapy-induced peripheral neuropathy (CIPN) is a dose-limiting side effect that largely remains an unresolved clinical issue, leading to long-term morbidity. This meta-analysis aimed to evaluate the efficacy and safety of Ganglioside-monosialic acid (GM1) in preventing CIPN.

**Methods:**

Systematic literature searches of PubMed, Web of Science, Embase, the Cochrane Central Register of Controlled Trials, and ClinicalTrials.gov were performed to identify randomized controlled trials and cohort studies that evaluated the efficacy of GM1 for preventing CIPN. Conventional meta-analysis with a random-effects model and trial sequential analysis (TSA) were performed.

**Results:**

A total of five studies involving 868 participants were included. The results showed that GM1 did not reduce the overall incidence of grade ≥ 2 CIPN when the common terminology criteria for adverse events (CTCAE) was used (OR 0.34, 95% CI 0.34–1.11). Subgroup analyses showed that GM1 could not reduce the risk of CTCAE grade ≥ 2 CIPN (OR 0.63, 95% CI 0.35–1.13) and neurotoxicity criteria of Debiopharm (DEB-NTC) grade ≥ 2 CIPN (OR 0.25, 95% CI 0.01–7.10) in oxaliplatin-treated patients, despite that GM1 was associated with a reduced risk of CTCAE grade ≥ 2 CIPN in the taxane subgroup of one study (OR 0.003, 95% CI 0.00–0.05). These results were confirmed by the sub-analysis of randomized controlled trials (RCTs). In TSA, the *z*-curve for the taxane subgroup crossed the upper trial sequential monitoring boundary (TSMB) but do not reach the required information size (RIS). The *z*-curves for the oxaliplatin subgroup remained in the nonsignificant area and did not reach the RIS. Further, GM1 did not influence the rate of response to chemotherapy and CTCAE grade ≥ 2 adverse events such as fatigue, nausea, diarrhea, and rash.

**Conclusions:**

GM1 seemed to be well-tolerated and did not influence the anti-cancer effects of chemotherapeutic agents. Although the data did not confirm the effectiveness of GM1 in preventing oxaliplatin-induced peripheral neuropathy, GM1 might be able to prevent taxane-induced peripheral neuropathy. More studies are required in different ethnic populations receiving taxane-based chemotherapy to confirm these findings.

**Supplementary Information:**

The online version contains supplementary material available at 10.1186/s12885-021-08884-4.

## Background

The incidence of cancer is still alarming globally [1] and despite breakthroughs in cancer treatment, chemotherapy is still an important cornerstone of cancer treatment [2]. Chemotherapy-induced peripheral neuropathy (CIPN) is a perturbing adverse effect for many cancer patients treated with chemotherapeutic agents, such as microtubule disruptors (taxanes, vinca alkaloids), platinum-based agents (cisplatin, oxaliplatin), epothilones (ixabepilone), and proteasome inhibitors (bortezomib) [3, 4]. It is believed to affect around 68 and 30% of patients treated using neurotoxic chemotherapy in the short and long term, respectively [5]. CIPN compromises the quality of daily life of these patients by impairing their sensory, motor, and autonomic functions [6], often causing chemotherapy dose reductions and discontinuations. Unfortunately, the pathogenetic mechanisms of CIPN genesis remain poorly understood. Available treatment options for CIPN are limited. To date, no agents have been recommended for the prevention of CIPN [7]. For these reasons, the identification of novel drugs for preventing CIPN is urgently needed in clinical practice.

Ganglioside-monosialic acid (GM1) is a monosialoglycosphingolipid mainly found in neurons and belongs to the family of gangliosides which are unique acidic glycolipids consisting of sphingosine, fatty acid, and sialic acid [8]. GM1 has been associated with essential functions in the processes of signal transduction, cell recognition, neurogenesis, and nerve development and differentiation [9–11]. In the 1980s, ganglioside treatment was found to be useful in the mitigation of vincristine-associated neuropathy, both in rabbit models and cancer patients [12, 13]. Preclinical animal models suggested that porcine GM1 could be effective in the prevention and treatment of paclitaxel-induced neuropathy [14]. Recently more and more researchers have started to focus on the possible efficacy of GM1 in preventing CIPN. A retrospective study showed that GM1 could significantly reduce the incidence of oxaliplatin-induced neuropathy [15]. However, the findings from two recent clinical trials have shown inconsistent results [16, 17]. Therefore, we performed this meta-analysis to evaluate the efficacy and safety of GM1 treatment for preventing CIPN.

## Methods

This study followed the Preferred Reporting Items for Systematic Reviews and Meta-Analyses (PRISMA) statement [18] and the Meta-analysis Of Observational Studies in Epidemiology (MOOSE) guidelines [19].

### Search strategy

Electronic databases including PubMed, Web of Science, Embase, the Cochrane Central Register of Controlled Trials (CENTRAL), and ClinicalTrials.gov were systematically searched from inception till June 12, 2021, without language restrictions. The search terms included *ganglioside-monosialic acid*, *GM1*, *ganglioside*, *monosialoganglioside*, and *chemotherapy-induced neuropathy*. Additionally, we checked the reference and citation lists of relevant publications for any unidentified studies. Details of our search strategy are presented in Supplementary Table S1. Retrieved study authors were contacted via e-mail or telephone for additional information when necessary.

### Inclusion and exclusion criteria

Studies were considered eligible if they were randomized controlled trials (RCTs), nonrandomized clinical studies, or observational cohort studies that compared GM1 with controls to prevent CIPN in cancer patients treated with neurotoxic chemotherapy. Control drugs were defined as no intervention, a placebo, or any drugs currently known not to relieve or prevent CIPN symptoms. Editorials, review articles, case reports, letters, and animal experimental studies were excluded.

### Data extraction and study endpoints

Two authors (SYW, XHB) independently extracted data from the eligible studies. Disagreements among authors were discussed and finally established by a third reviewer (WAZ). The extracted data included general study characteristics (first author’s name, publication year, study sites, study design, trial registry numbers, study duration); baseline patient demographics (age, sample size, types of malignancy, chemotherapy regimen); interventions (dose of GM1, controls); and endpoints of interest. Our primary endpoints were the incidence of CIPN measured with the common terminology criteria for adverse events (CTCAE) version 4.0 and neurotoxicity criteria of Debiopharm (DEB-NTC) [20]. Our secondary endpoints were the following: (1) response rate to chemotherapy (Response Evaluation Criteria in Solid Tumors [RECIST]) [21], (2) adverse events related to GM1, and (3) proportion of patients that drop out of chemotherapy.

### Quality assessment for the included studies

Two authors (SYW, CXG) independently evaluated the risk of bias in each study. Disagreements were resolved via consultation with another author (WAZ). The revised Cochrane risk-of-bias tool for randomized trials (RoB 2.0) [22] was used for RCTs, and Risk of Bias In Non-randomized Studies of Interventions (ROBINS-I tool) [23] was used for observational studies.

### Statistical analysis

All statistical methods followed the principles outlined in the Cochrane Handbook for Systematic Reviews of Interventions [24]. All data were combined as pooled odds ratios (ORs) with 95% confidence intervals (CIs) using the Mantel-Haenszel statistical method. Random-effects models were used to pool the data across all outcomes to produce more conservative estimates [25]. Following the guidelines provided by the Cochrane Handbook, non-RCTs were included due to the small number of RCTs available in the area of interest. Heterogeneity was evaluated using *I*^2^ statistics (> 75% indicating high heterogeneity) and Q statistics (with a significance level set at *P* = 0.10) [26]. Subgroup analyses were conducted for the primary endpoints with consideration of potential sources of heterogeneity (effect modifiers): type of chemotherapy drugs used, age, gender. Sensitivity analyses for the primary endpoints were also performed by omitting the non-randomized studies to determine the robustness of our results. All meta-analyses were conducted using Stata SE version 12.0 (Stata Corp., College Station, TX, USA) and RevMan version 5.3 (Nordic Cochrane Center, Cochrane Collaboration). All statistical tests were two-tailed, and *P* < 0.05 was considered statistically significant, except otherwise specified.

*Post-hoc* trial sequential analysis (TSA) was also performed for our primary endpoints. Two-sided *z*-score thresholds were adjusted using the O’Brien-Fleming α-spending function with a power of 80% and with 2-sided 5% type 1 error to constructing trial sequential monitoring boundaries (TSMB) [27]. Information sizes were estimated from all sample sizes. Control arm incidences were calculated using event rates from all included studies, heterogeneity correction was model variance-based [27]. TSA was performed using the TSA software version 0.9.5.10 Beta (Copenhagen trial unit, Denmark).

## Results

### Study selection

**Fig. 1:**
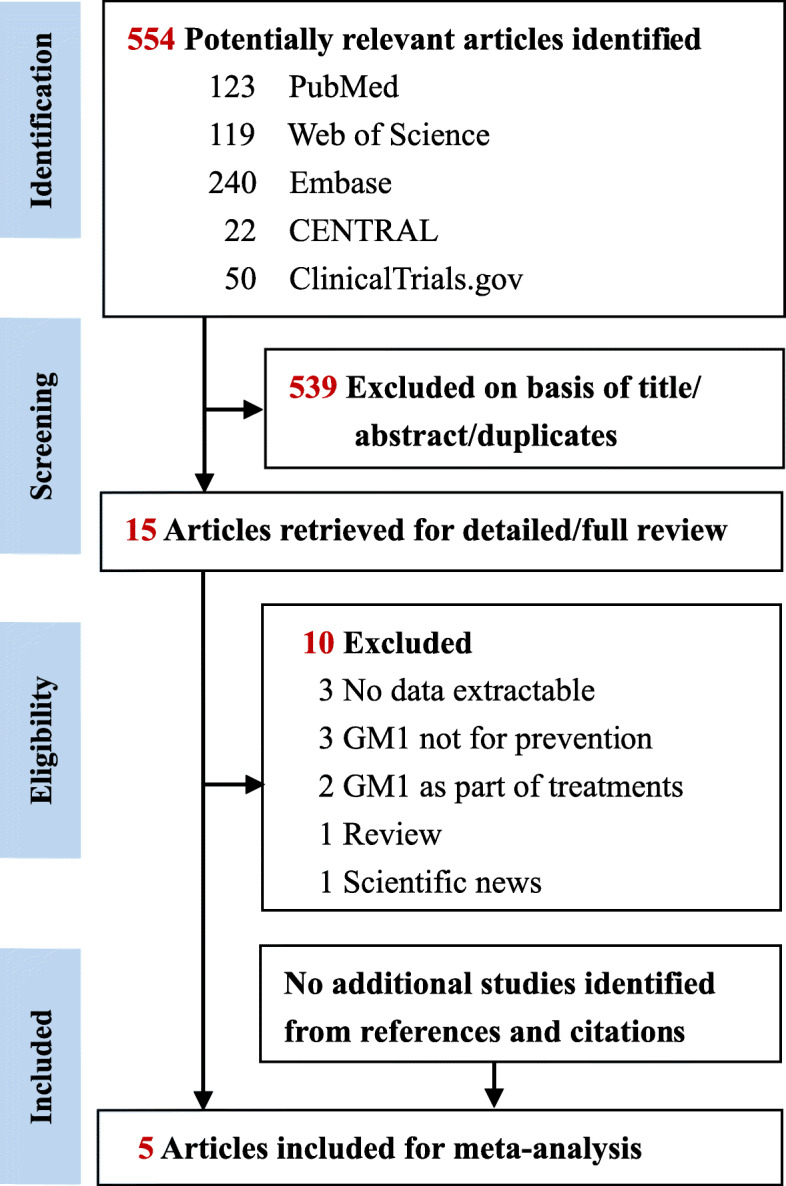
Flowchart of the literature search and study selection

The systematic literature search yielded 554 records (Supplementary Table S1). After the titles, abstracts, and duplicates were screened, 15 articles were considered potentially relevant. Of these, ten articles were excluded because of non-extractable data (*n* = 3), lacked assessment on the prevention of CIPN (n = 3), absence of appropriate treatment group (*n* = 2), and unsuitable article type (review article, *n* = 1; scientific news, n = 1). Thus, four RCTs [16, 17, 28, 29] and one retrospective cohort study [15] were included after full-text review for further analysis. The flowchart of the search strategy and study selection is illustrated in Fig. 1.

### Characteristics and quality assessment of included studies

The general characteristics of all included studies are summarized in Table 1. These five studies involved 868 patients (413 patients in the GM1 group and 455 patients in the control group). All of them were conducted in China and published between 2012 and 2020. Sample size, types of cancer, follow-up duration were well-balanced and comparable in each study between the intervention and control group (Table 1).

**Table 1:** Characteristics of included studies

Study	study design	Type of malignancy	Chemotherapy regimen	Participants				GM1 regimen	Controls	Mean cumulative dose of chemotherapy (GM1 vs. control)	Endpoints	Study Duration
				age, yr (mean/median)	age range	GM1	Control					
Chen et al., 2012	Retrospective	colorectal cancer	mFOLFOX6, FOLFOX4, XELOX	53.5 (median)	36–75	114	164	40 mg*4/cycle	None	oxaliplatin, 1120 vs. 960 mg/m^2^	CIPN (CTCAE); RECIST; CD	24.6 months follow-up
Zhu et al., 2013	RCT	gastrointestinal cancer	FOLFOX4, XELOX	54.96 (mean)	21–74	60	60	100 mg*3/cycle	None	oxaliplatin, 692.08 vs. 740.83 mg/m^2^	CIPN (CTCAE); RECIST	over 3 months after chemotherapy
Cao et al., 2014	RCT	gastrointestinal cancer	FOLFOX6, XELOX	56 (median)	31–72	38	30	40 mg*3/cycle	Vitamin B12	NR	CIPN (DEB-NTC); RECIST; AEs	>12w
Su et al., 2020 (NCT02468739)	RCT (multicenter)	breast cancer	EC-P, EC-D, DC	44.5 (median)	23–74	103	103	80 mg*3/cycle	Placebo	paclitaxel, 942.67 vs. 954.29 mg/m^2^; Docetaxel, 525.31 vs. 501.81 mg/m^2^	CIPN (CTCAE); AEs	12 months after completion of chemotherapy
Wang et al., 2020 (NCT02251977)	RCT (multicenter)	colorectal cancer	mFOLFOX6, XELOX	52.6 (mean)	> 18	98	98	80 mg*5/cycle	Placebo	NR	CIPN (CTCAE, DEB-NTC); AEs; CD	48 months after chemotherapy

*AEs* Adverse events, *CD* Chemotherapy dropout, *CIPN* Chemotherapy-induced peripheral neuropathy, *CTCAE* Common terminology criteria for adverse events, *DC* Docetaxel and cyclophosphamide, *DEB-NTC* Neurotoxicity criteria of Debiopharm, *EC-P*, Epirubicin and cyclophosphamide followed by paclitaxel, *EC-D* Epirubicin and cyclophosphamide followed by docetaxel, *mFOLFOX6* Modified FOLFOX6, *NR* Not reported, *RCT* Randomized controlled trial, *RECIST* Response evaluation criteria in solid tumors

**Fig. 2:**
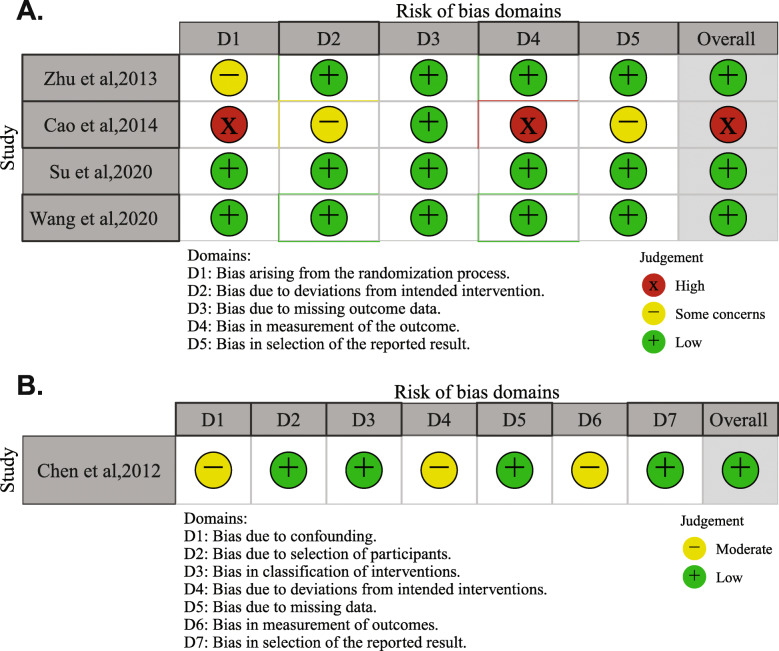
Risk-of-bias assessment of included RCTs using the revised Cochrane risk-of-bias tool for randomized trials (RoB 2.0) (A) and of one cohort study using Risk of Bias In Non-randomized Studies of Interventions (ROBINS-I tool) (B)

Using the risk of bias tool for RCTs (RoB 2.0), two RCTs [16, 17] were found to have a low risk of bias because they are well-designed randomized, multicenter, double-blind, placebo-controlled trial, one RCT [29] was also considered to have a low risk of bias despite we had some concerns about its randomization process, while the other RCT [28] had a high risk of bias, due to lack of information on the randomization process and bias in measurement of the outcomes (Fig. 2A). The included observational study [15] had a low risk of bias according to the ROBINS-I (Fig. 2B).

### Primary endpoints

#### Incidence of CIPN (CTCAE)

**Fig. 3:**
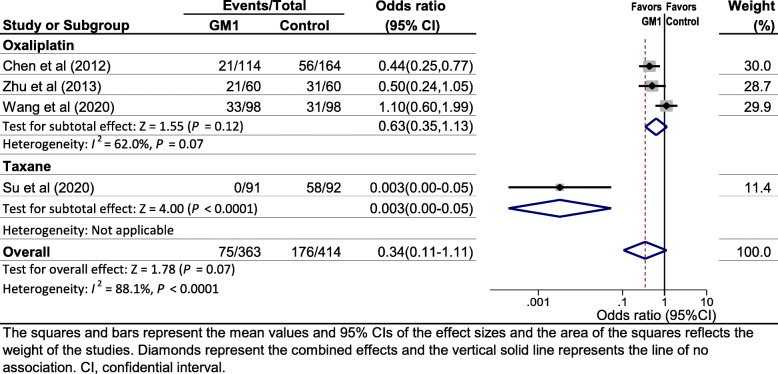
Forest plot displaying a random-effects meta-analysis of the effect of GM1 on the incidence of grade ≥ 2 chemotherapy-induced peripheral neuropathy using the common terminology criteria for adverse events (CTCAE)

Four studies [15–17, 29] reported data regarding the incidence of grade ≥ 2 CIPN using the CTCAE measure. Of the four studies, three studies [15, 16, 29] used the oxaliplatin regimen while only one study [17] used the taxanes regimen. Pooled data showed a tendency to reduce the risk of grade ≥ 2 CIPN (CTCAE), but was not statistically significant (OR 0.34, 95% CI 0.11–1.11, *P* = 0.07; Fig. 3), and substantial heterogeneity was observed (*I*^2^ = 88.1%, *P* < 0.0001; Fig. 3).

#### Incidence of CIPN (DEB-NTC)

**Fig. 4:**
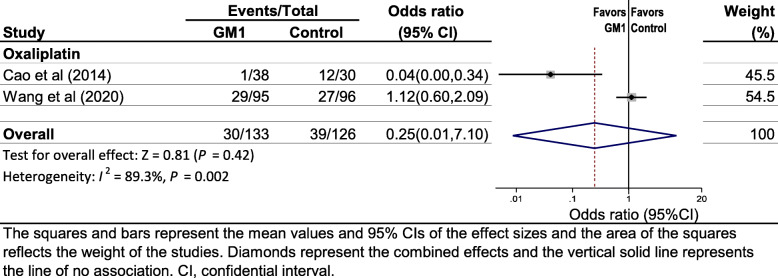
Forest plot displaying a random-effects meta-analysis of the effect of GM1 on the incidence of grade ≥ 2 oxaliplatin-induced peripheral neuropathy using the Neurotoxicity criteria of Debiopharm (DEB-NTC)

Two studies [16, 28] reported data on the incidence of grade ≥ 2 CIPN measured with the Neurotoxicity criteria of Debiopharm (DEB-NTC), which was an oxaliplatin-specific neuropathy grading scale [30]. Pooled data showed that GM1 was not associated with a lower incidence of grade ≥ 2 CIPN (OR 0.25, 95% CI 0.01–7.10, *P* = 0.42) when compared with controls. Heterogeneity was substantial (*I*^2^ = 89.3%, *P* = 0.002; Fig. 4).

### Secondary endpoints

#### Objective response rates to chemotherapy (RECIST)

**Fig. 5:**
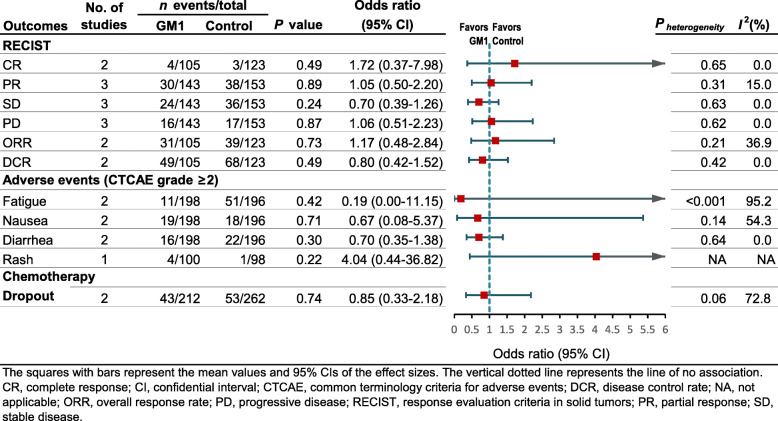
Summary of RECIST and safety data related to GM1

Pooled data from three studies [15, 28, 29] that assessed the objective response rates to chemotherapy, including complete response (CR), partial response (PR), stable disease (SD), progressive disease (PD), overall response rate (ORR), and disease control rate (DCR), showed that GM1 did not influence any of the above-mentioned response rate parameters, indicating that GM1 could not affect the antineoplastic activity of chemotherapeutic agents. The heterogeneity for those parameters was relatively low (Fig. 5).

#### Incidence of adverse events (CTCAE)

The incidence of adverse events was investigated using the CTCAE measure. Two studies [16, 17] reported data on the risk of grade ≥ 2 fatigue, nausea, and diarrhea, and these data were pooled for meta-analysis. GM1 did not influence the incidence of grade ≥ 2 fatigue (OR 0.19, 95% CI 0.00–11.15, *P* = 0.42), the risk of grade ≥ 2 nausea (OR 0.67, 95% CI 0.08–5.37, *P* = 0.71), or the risk of grade ≥ 2 diarrhea (OR 0.70, 95% CI 0.35–1.38, *P* = 0.30). There was low heterogeneity in grade ≥ 2 diarrhea (*I*^2^ = 0.0%, *P* = 0.64); however, the heterogeneity in grade ≥ 2 fatigue was high (*I*^2^ = 95.2%, *P* < 0.001), and the heterogeneity in grade ≥ 2 nausea was moderate (*I*^2^ = 54.3%, *P* = 0.14, Fig. 5). Only one study [17] reported on the incidence of taxane-associated rash and no statistically significant difference was observed in grade ≥ 2 rash (OR 4.04, 95% CI 0.44–36.82, *P* = 0.22, Fig. 5).

#### Chemotherapy dropout

Pooled data from two studies [15, 16] that assessed chemotherapy dropout suggested that GM1 did not influence the risk of chemotherapy dropout (OR 0.85, 95% CI 0.33–2.18, *P* = 0.74) with moderate heterogeneity (*I*^2^ = 72.8%, *P* = 0.06; Fig. 5).

### Subgroup analyses

Since no significant effect was observed on the overall incidence of CTCAE grade ≥ 2 CIPN in the primary analysis, we further conducted subgroup analysis to explore the effect of the type of chemotherapy drugs on the results. For the endpoint of CTCAE grade ≥ 2 CIPN, GM1 was associated with a lower risk of taxane-induced peripheral neuropathy (OR 0.003, 95% CI 0.00–0.05) but did not reduce the risk of oxaliplatin-induced peripheral neuropathy (OR 0.63, 95% CI 0.35–1.13) (Fig. 3). In addition, subgroup analysis stratified by the type of chemotherapy drugs resulted in much smaller heterogeneity (Fig. 3). However, subgroup analyses stratified by other factors such as age, gender were not conducted because no sufficient data were available.

### Sensitivity analysis

Sensitivity analyses were conducted for the incidence of CTCAE grade ≥ 2 CIPN by removing the only retrospective study [15], and the results are presented in Supplementary Fig. S1. When only RCTs were included, GM1 was not associated with reduced risk of CTCAE grade ≥ 2 oxaliplatin-induced peripheral neuropathy (OR 0.77, 95% CI 0.36–1.64), which was consistent with the previous main analysis of this study.

### Trial sequential analysis (TSA) for the incidence of CIPN

**Fig. 6:**
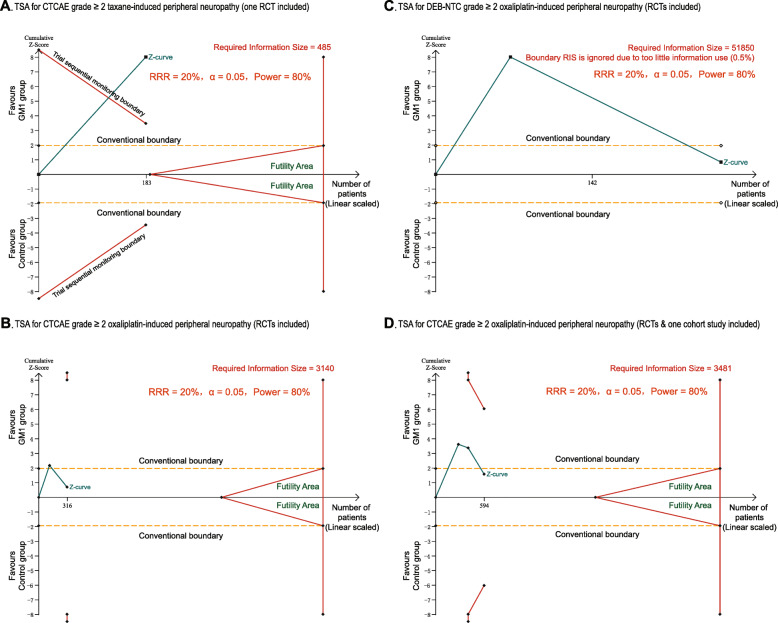
TSA for taxane-induced peripheral neuropathy showing that the cumulative *z*-curve (dark green line) has crossed the upper (superiority) TSMB (red line) for statistical significance, but does not reach the required information size (RIS) (vertical red line) (**A**). TSA for oxaliplatin-induced peripheral neuropathy showing that the cumulative *z*-curve (dark green line) has crossed neither any TSMB (red line) for benefit nor the RIS, finally stays in the nonsignificant area (**B, C, D**). Of note, the result of the DEB-NTC measure shows that the cumulative sample size in the included studies (133 + 126 = 259) was far less than the RIS of 51,850, indicating that the effect of GM1 on oxaliplatin-induced peripheral neuropathy was far from conclusive

The panels (Fig. 6A-D) showed the relations between the *z*-curves, conventional boundaries, TSMBs, and RIS. The *z*-curves from the first meta-analysis that assessed the GM1 in the subgroup of taxane crossed both the conventional boundary and the upper (superiority) TSMB, but not RIS, after only one RCT was included (Fig. 6A). The two RCTs meta-analyses which evaluated GM1 in the subgroup of oxaliplatin using CTCAE and DEB-NTC showed that it did not achieve sufficient sample size to verify a minor effect, as the *z*-curves temporarily crossed the conventional boundary, and finally returned to nonsignificant values (Fig. 6B-C). Similarly, the results of TSA with all three studies [15, 16, 29] which evaluated GM1 for the subgroup of oxaliplatin using CTCAE showed no difference with the two RCTs meta-analysis using CTCAE, as the *z*-curve also returned to nonsignificant values after crossing the conventional boundary temporarily (Fig. 6D).

## Discussion

This meta-analysis included four RCTs and one retrospective cohort study, comprising a total of 868 patients, and evaluated the efficacy and safety of GM1 in preventing CIPN caused by the two most prominent types of neurotoxic antineoplastic agents, namely taxanes and oxaliplatin. Our analyses suggest that GM1 did not reduce the overall incidence of CIPN; however, GM1 might have different effects on CIPN based on different chemotherapy drugs. Also, GM1 was well-tolerated and did not influence the anti-tumor activity of chemotherapeutic agents (Fig. 5).

In this study, the efficacy of GM1 on CIPN prevention was assessed using two tools - the CTCAE and DEB-NTC. The CTCAE is a common tool for assessing CIPN symptoms and other chemotherapy-related adverse events, consisting of 5 grades (CTCAE grade 1–5). In contrast, the DEB-NTC is a specific tool for oxaliplatin-induced neurotoxicity assessment, consisting of 3 grades (DEB-NTC grade 1–3) (Supplementary Table S2). Both CTCAE and DEB-NTC are clinician-reported measures. Patient-reported outcome (PRO) measures such as European Organization for Research and Treatment of Cancer Quality of Life Questionnaire-Chemotherapy Induced Peripheral Neuropathy 20 (EORTC QLQ CIPN-20) and Functional Assessment of Cancer Therapy/Gynecologic Oncology Group-Neurotoxicity (FACT/GOG-Ntx) were not used in our analysis because of lack of sufficient data. Although some discrepancies in the explanation of clinical and PRO measures were observed, Jennifer et al. [31] found that the association between QLQ CIPN-20 scores and CTCAE grades was strong. Of note, the QLQ CIPN-20 score was not significantly different for oxaliplatin-induced neuropathy between GM1 and the control group in the study by Wang et al. [16], which was consistent with the results of our meta-analysis measured by CTCAE and DEB-NTC. Similarly, in the study of Su et al. [17], both FACT-Ntx scores and CTCAE showed the effectiveness of GM1 in preventing the neurotoxicity of taxanes. Thus, we believe that our results are robust despite the lack of PRO measures in our analyses.

Findings of the present study obtained different results regarding the effectiveness of GM1 on the incidence of neurotoxicity of taxanes and oxaliplatin in the subgroup analyses. There are two potential explanations for these results. First, the toxicity profile differs among different drugs. Pachman et al. [32] found that oxaliplatin-induced neurotoxicity deteriorated after the completion of treatment but began to improve 3 months after the treatment; unlike, paclitaxel-induced neuropathy began improving immediately after chemotherapy cessation. This means that oxaliplatin-induced neuropathy might be more severe and refractory to conventional treatment than taxanes. Second, each anti-cancer agent induces CIPN through different mechanisms; for example, taxanes damage neuronal axons by causing stabilization of microtubules, while platinum derivatives accumulate in the cell bodies of sensory nerves, and react with DNA to form both intrastrand and interstrand cross-links [33]. The underlying mechanism of GM1 and the reason why GM1 seems to be more useful in taxane-induced neurotoxicity remains to be fully elucidated.

The latest clinical guideline for the prevention and management of CIPN did not recommend any agents for preventing CIPN [7]. In this updated guideline [7], the evidence for the efficacy of GM1 was deemed preliminary because the taxane-induced neuropathy in the study by Su et al. [17] was almost totally resolved 3 months after the completion of taxane therapy, which was faster than other trials [34]. In addition, our TSA finding suggested that the evidence for GM1 should be considered encouraging but inconclusive until further confirmatory studies are performed. However, this present study still sheds light on solving the challenging issues confronted by cancer patients treated with taxanes. In the future, more trials including patients from both western and eastern nationalities are needed. Currently, two clinical trials registered at ClinicalTrials.gov (NCT02500810, NCT04222790) evaluating the effectiveness of GM1 to prevent albumin-bound paclitaxel neurotoxicity are still underway and their results are awaited. We could recommend global multisite studies if the results of the two studies (NCT02500810, NCT04222790) are encouraging. On the other hand, TSA findings on the GM1 for oxaliplatin suggest that the prospect for its prophylactic use in oxaliplatin-treated patients looks uncertain. The official reports of the ongoing RCTs (NCT02024412, NCT02024438), which aimed to evaluate GM1 in oxaliplatin-treated patients and started recruitment more than 7 years ago, are still awaited.

Notably, the use of GM1 could lead to human autoimmune neuropathy in rare cases, leading to ganglioside-associated Guillain-Barré syndrome (GBS) [35], a serious complication from gangliosides use, which usually manifests as limb weakness and presents with rapidly progressive paralysis; often needing artificial ventilation [36]. A case series of seven patients who suffered from ganglioside-associated GBS was reported in northeast China [37]; however, the actual incidence of GBS thus far is unknown due to the widespread use of GM1 in China. Besides, the molecular pathogenesis of this syndrome was clarified by Yuki et al. [38] who established a disease model for GBS by sensitization with GM1 and confirmed the relationship between anti-GM1 antibody and GBS. Most countries, except China, withdrew gangliosides from the therapeutic market [39]. In China, the indications of GM1 include stroke, traumatic brain or spinal cord injury, and Parkinson’s Disease. Although there are no reports about the cases of ganglioside-associated GBS in the studies included in our meta-analysis, GM1 should be used only when necessary.

To our knowledge, this is the first meta-analysis focused on identifying the effectiveness and safety of GM1 on CIPN based on the latest well-designed multicenter RCTs [16, 17]. The strengths of this study were as follows: first, the latest risk-of-bias tools including RoB2.0 and ROBINS-I were adopted and provided a comprehensive evaluation on the quality of the included studies. Second, the implementation of a TSA methodology explored the current status of evidence for the effectiveness of prophylactic use of GM1 in cancer patients.

However, this study also had some potential limitations. First, all the included studies were conducted in China, so the results may be influenced by patient selection factors such as Chinese ethnicity. Second, the inclusion of one retrospective study in the meta-analysis may have contributed to a higher risk of bias. Third, due to the limited number of studies included in each analysis, publication bias could not be assessed. Fourth, more information to determine whether the benefits of GM1 outweigh its clinical risks (i.e., GM1-induced GBS that led to its removal from many markets) is needed, and the cost-benefit for GM1 use remains to be determined because no reports of any case of GBS were included in this meta-analysis. Fifth, the reliability and validity of the outcome measures (CTCAE and DEB-NTC) in this present study should be validated. In real-world studies, to achieve a comprehensive measure of CIPN, both clinical and PRO measures should be used [40], which should be followed in further studies on CIPN caused by the two types of chemotherapy agents or any other agent.

## Conclusions

GM1 seems to be well-tolerated and does not influence the anti-cancer effects of chemotherapeutic agents. Our data did not confirm the effectiveness of GM1 in preventing oxaliplatin-induced peripheral neuropathy with limited evidence; however, GM1 demonstrated the potential to prevent taxane-induced peripheral neuropathy. More well-designed studies are recommended in different ethnic populations receiving taxane-based chemotherapy to validate these findings.

## Supplementary Information

**Additional file 1: Supplementary Table S1.** Search strategy in this meta-analysis.

**Additional file 2: Supplementary Table S2.** Criteria of neurotoxicity according to the NCI-CTCAE ver. 4.0 and DEB-NTC scales.

**Additional file 3: Supplementary Fig. S1.** Sensitivity analysis of forest plot displaying a random-effects meta-analysis of the effect of GM1 on the incidence of CTCAE grade ≥ 2 when only RCTs were included.

## Data Availability

All data generated or analyzed during this study are included in this published article. No additional data are available.

## Abbreviations

CENTRAL: The Cochrane Central Register of Controlled Trials
CI: Confidential interval
CIPN: Chemotherapy-induced peripheral neuropathy
CR: Complete response
CTCAE: Common terminology criteria for adverse events
DCR: Disease control rate
DEB-NTC: Neurotoxicity criteria of Debiopharm
GBS: Guillain-Barré syndrome
GM1: Ganglioside-monosialic acid
NA: Not applicable
OR: Odds ratio
ORR: Overall response rate
PD: Progressive disease
PR: Partial response
RCT: Randomized controlled trial
RECIST: Response evaluation criteria in solid tumors
RIS: Required information size
SD: Stable disease
TSA: Trial sequential analysis
TSMB: Trial sequential monitoring boundary
