# Supplementary material for: Ganglioside-monosialic acid (GM1) for prevention of chemotherapy-induced peripheral neuropathy: a meta-analysis with trial sequential analysis
Source: BMC Cancer. 2021 Nov 2;21:1173. doi: 10.1186/s12885-021-08884-4 (PMC8564974; doi:10.1186/s12885-021-08884-4)
Supplement: Supplementary file 1 — Additional file 1: Supplementary Table S1. Search strategy in this meta-analysis. [file 12885_2021_8884_MOESM1_ESM.doc]

**Supplementary Table S1**: Search strategies in this meta-analysis

| Database | Search strategy | Results |
| --- | --- | --- |
| PubMed | (ganglioside* OR monosialoganglioside OR GM1) AND (cisplatin OR carboplatin OR oxaliplatin OR platinum OR paclitaxel OR docetaxel OR taxane* OR vinorelbine OR vincristine OR vinblastine OR vindesine OR vinca alkaloid* OR doxorubicin OR ifosfamide OR 5-Fluorouracil OR 5-FU OR methotrexate OR bortezomib OR thalidomide OR lenalidomide OR ixabepilone OR eribulin mesylate OR suramin) AND (chemotherapy OR neurotoxicity OR neuropathy OR toxicity) | 123 |
| Web of Science | TOPIC: (ganglioside* OR monosialoganglioside OR GM1) AND TOPIC: (cisplatin OR carboplatin OR oxaliplatin OR platinum OR paclitaxel OR docetaxel OR taxane* OR vinorelbine OR vincristine OR vinblastine OR vindesine OR vinca alkaloid* OR doxorubicin OR ifosfamide OR 5-Fluorouracil OR 5-FU OR methotrexate OR bortezomib OR thalidomide OR lenalidomide OR ixabepilone OR eribulin mesylate OR suramin) AND TOPIC: (chemotherapy OR neurotoxicity OR neuropathy OR toxicity)  Timespan: All years. Databases: Web of Science Core Collection, BIOSIS, CSCD, DIIDW, INSPEC, KJD, MEDLINE, RSCI, SCIELO.  Search language=Auto. | 119 |
| Embase | (ganglioside* OR 'monosialoganglioside'/exp OR monosialoganglioside OR gm1) AND (('cisplatin'/exp OR cisplatin OR 'carboplatin'/exp OR carboplatin OR 'oxaliplatin'/exp OR oxaliplatin OR 'platinum'/exp OR platinum OR 'paclitaxel'/exp OR paclitaxel OR 'docetaxel'/exp OR docetaxel OR taxane* OR 'vinorelbine'/exp OR vinorelbine OR 'vincristine'/exp OR vincristine OR 'vinblastine'/exp OR vinblastine OR 'vindesine'/exp OR vindesine OR 'vinca'/exp OR vinca) AND alkaloid* OR 'doxorubicin'/exp OR doxorubicin OR 'ifosfamide'/exp OR ifosfamide OR '5 fluorouracil'/exp OR '5 fluorouracil' OR '5 fu'/exp OR '5 fu' OR 'methotrexate'/exp OR methotrexate OR 'bortezomib'/exp OR bortezomib OR 'thalidomide'/exp OR thalidomide OR 'lenalidomide'/exp OR lenalidomide OR 'ixabepilone'/exp OR ixabepilone OR 'eribulin mesylate'/exp OR 'eribulin mesylate' OR (('eribulin'/exp OR eribulin) AND ('mesylate'/exp OR mesylate)) OR 'suramin'/exp OR suramin) AND ('chemotherapy'/exp OR chemotherapy OR 'neurotoxicity'/exp OR neurotoxicity OR 'neuropathy'/exp OR neuropathy OR 'toxicity'/exp OR toxicity) | 240 |
| Cochrane Central Register of Controlled Trials (CENTRAL) | (ganglioside* OR monosialoganglioside OR GM1):ti,ab,kw AND (cisplatin OR carboplatin OR oxaliplatin OR platinum OR paclitaxel OR docetaxel OR taxane* OR vinorelbine OR vincristine OR vinblastine OR vindesine OR vinca alkaloid* OR doxorubicin OR ifosfamide OR fluorouracil OR FU OR methotrexate OR bortezomib OR thalidomide OR lenalidomide OR ixabepilone OR eribulin mesylate OR suramin):ti,ab,kw AND (chemotherapy OR neurotoxicity OR neuropathy OR toxicity):ti,ab,kw" (Word variations have been searched) | 22 |
| ClinicalTrials.gov | ganglioside* OR monosialoganglioside OR GM1 | 50 |
