# Supplementary material for: Ganglioside-monosialic acid (GM1) for prevention of chemotherapy-induced peripheral neuropathy: a meta-analysis with trial sequential analysis
Source: BMC Cancer. 2021 Nov 2;21:1173. doi: 10.1186/s12885-021-08884-4 (PMC8564974; doi:10.1186/s12885-021-08884-4)
Supplement: Supplementary file 2 — Additional file 2: Supplementary Table S2. Criteria of neurotoxicity according to the NCI-CTCAE ver. 4.0 and DEB-NTC scales. [file 12885_2021_8884_MOESM2_ESM.docx]

Supplementary Table S2: Criteria of neurotoxicity according to the NCI-CTCAE ver. 4.0 and DEB-NTC scales [[1](#_ENREF_1)]

| Grade | NCI-CTCAE | DEB-NTC |
| --- | --- | --- |
| 1 | Asymptomatic; loss of deep tendon reflexes or paresthesia (including tingling), but not interfering with function | Within 7 days |
| 2 | Sensory alteration or paresthesia (including tingling) interfering with function, but not interfering with ADL | More than 7 days |
| 3 | Sensory alteration or paresthesia interfering with ADL | Functional impairment interfering with ADL |
| 4 | Disability | - |
| 5 | Death | - |

ADL: activities of daily living

1. Inoue N, Ishida H, Sano M, Kishino T, Okada N, Kumamoto K, Ishibashi K: **Discrepancy between the NCI-CTCAE and DEB-NTC scales in the evaluation of oxaliplatin-related neurotoxicity in patients with metastatic colorectal cancer**. *International journal of clinical oncology* 2012, **17**(4):341-347.
