# Supplementary material for: Ganglioside-monosialic acid (GM1) for prevention of chemotherapy-induced peripheral neuropathy: a meta-analysis with trial sequential analysis
Source: BMC Cancer. 2021 Nov 2;21:1173. doi: 10.1186/s12885-021-08884-4 (PMC8564974; doi:10.1186/s12885-021-08884-4)
Supplement: Supplementary file 3 — Additional file 3: Supplementary Fig. S1. Sensitivity analysis of forest plot displaying a random-effects meta-analysis of the effect of GM1 on the incidence of CTCAE grade ≥ 2 when only RCTs were included. [file 12885_2021_8884_MOESM3_ESM.docx]

Supplementary Figure S1: Sensitivity analysis of forest plot displaying a random-effects meta-analysis of the effect of GM1 on the incidence of CTCAE grade ≥2 when RCTs included only.
